# Ultrasound-Detected Ulnar Nerve Entrapment With Normal Electrodiagnostic Studies Following Thoracic Outlet Decompression: A Case Report

**DOI:** 10.7759/cureus.108345

**Published:** 2026-05-06

**Authors:** Waseem Syed, Gurinder Singh, Justin McGinnis, Stephen Westfall, Joe Bhagratie

**Affiliations:** 1 College of Medicine, Lake Erie College of Osteopathic Medicine, Bradenton, USA; 2 Orthopedics, Baptist Medical Center Jacksonville, Jacksonville, USA

**Keywords:** hydrodissection, thoracic outlet syndrome, tinel’s test, ulnar nerve, ultrasound

## Abstract

We report the case of a 38-year-old female who developed persistent right upper extremity paresthesia, neuropathic pain, and ulnar-sided sensory disturbance following surgical decompression for thoracic outlet syndrome (TOS). Despite clinically significant cubital tunnel tenderness and ultrasound evidence of focal ulnar nerve impingement, electrodiagnostic studies, including nerve conduction studies (NCS) and electromyography (EMG), were normal. The patient underwent ultrasound-guided ulnar nerve hydrodissection with corticosteroid and dextrose solution as part of conservative management. This case underscores that cubital tunnel syndrome should not be excluded solely on the basis of normal electrodiagnostic studies, particularly when clinical and imaging findings are suggestive.

## Introduction

Cubital tunnel syndrome is the second most common entrapment neuropathy of the upper extremity after carpal tunnel syndrome [1]. It results from compression or traction of the ulnar nerve at the elbow, most commonly within the cubital tunnel. Clinical manifestations include paresthesia in the ulnar distribution, hand weakness, intrinsic muscle atrophy, and symptoms exacerbated by elbow flexion, particularly during prolonged positioning such as phone use or sleeping with a flexed elbow [2]. In more advanced cases, patients may demonstrate decreased grip strength, impaired dexterity, nd clawing of the fourth and fifth digits.

Diagnosis typically relies on a combination of clinical examination and electrodiagnostic testing, including nerve conduction studies (NCS) and electromyography. However, the sensitivity of electrodiagnostic testing is limited in the early stages of the disease and may fail to detect dynamic or intermittent compression [3]. This limitation is particularly relevant in patients whose symptoms are position-dependent or who have mild demyelination without significant axonal loss. As a result, imaging modalities such as high-resolution ultrasound have emerged as valuable adjuncts, allowing for dynamic assessment of nerve enlargement, subluxation, or focal compression.

Persistent postoperative symptoms are often multifactorial in nature, including nerve traction, perineural scarring, inflammation, and altered biomechanics after thoracic outlet decompression. Thoracic outlet syndrome (TOS) decompression surgery can improve neurovascular symptoms but may also unmask or exacerbate distal compressive neuropathies due to changes in neural tension and limb kinematics [4]. Additionally, postoperative changes in posture and muscle recruitment patterns may further contribute to symptom persistence or evolution.

This case illustrates persistent ulnar neuropathic symptoms following TOS surgery with normal EMG/NCS findings, emphasizing the diagnostic challenges in such presentations. It also highlights the importance of a multimodal diagnostic approach and the potential role of ultrasound-guided interventions in both confirming the diagnosis and providing symptomatic relief.

Given the rarity of TOS and the relative scarcity of reported cases demonstrating discordance between clinical symptoms and electrodiagnostic findings in this context, this case offers valuable insight that may help guide future diagnostic and management strategies.

## Case presentation

A 38-year-old female with a history of TOS underwent anterior and middle scalene resection on 12/11/2025 for neurogenic compression. She presented with persistent right upper extremity sensory disturbances postoperatively. Her chief complaint was persistent right elbow paresthesia with ulnar-sided hand numbness, intermittent neuropathic pain, and cold sensitivity after thoracic outlet decompression surgery. Immediately after surgery, the patient developed new-onset right elbow paresthesia that was not present preoperatively. During the first four to five weeks postoperatively, the patient experienced intermittent neuropathic pain radiating distally from the elbow into the ulnar aspect of the hand, accompanied by marked cold hypersensitivity. She reported partial symptomatic relief with gabapentin, naproxen, lidocaine patches, and heat therapy. Over time, neuropathic pain improved; however, persistent localized paresthesia and mechanical sensitivity at the elbow remained. She described a “jolting” sensation with minor contact over the posterior-medial elbow. Preoperatively, she had intermittent digit paresthesia during heavy manual labor, which improved following surgery. However, she also noted a decrease in grip strength from about 100 pounds to 50 pounds. She denied diabetes, thyroid disease, prior upper extremity trauma, or systemic neuropathic conditions.

Physical examination showed marked tenderness over the right cubital tunnel, positive Tinel sign at the medial elbow, 4/5 strength in both upper extremities, and grossly intact sensation to both light touch and pinprick. Reflexes were noted to be 2+ and symmetric. No intrinsic hand muscle atrophy was noted upon initial inspection.

**Figure 1:**
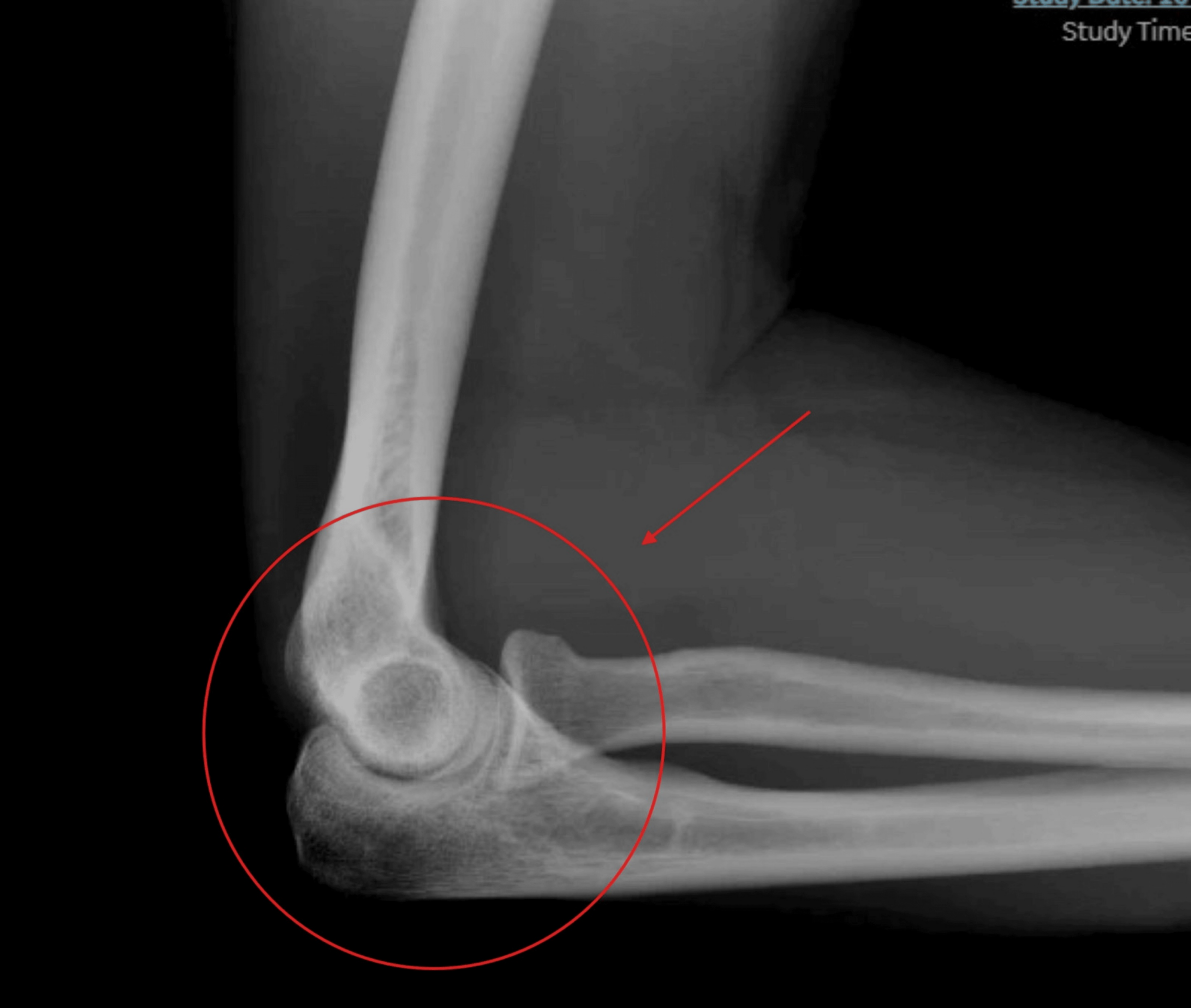
Lateral radiograph of the right elbow demonstrating normal osseous alignment The highlighted section demonstrates normal osseous alignment of the bone.

**Figure 2:**
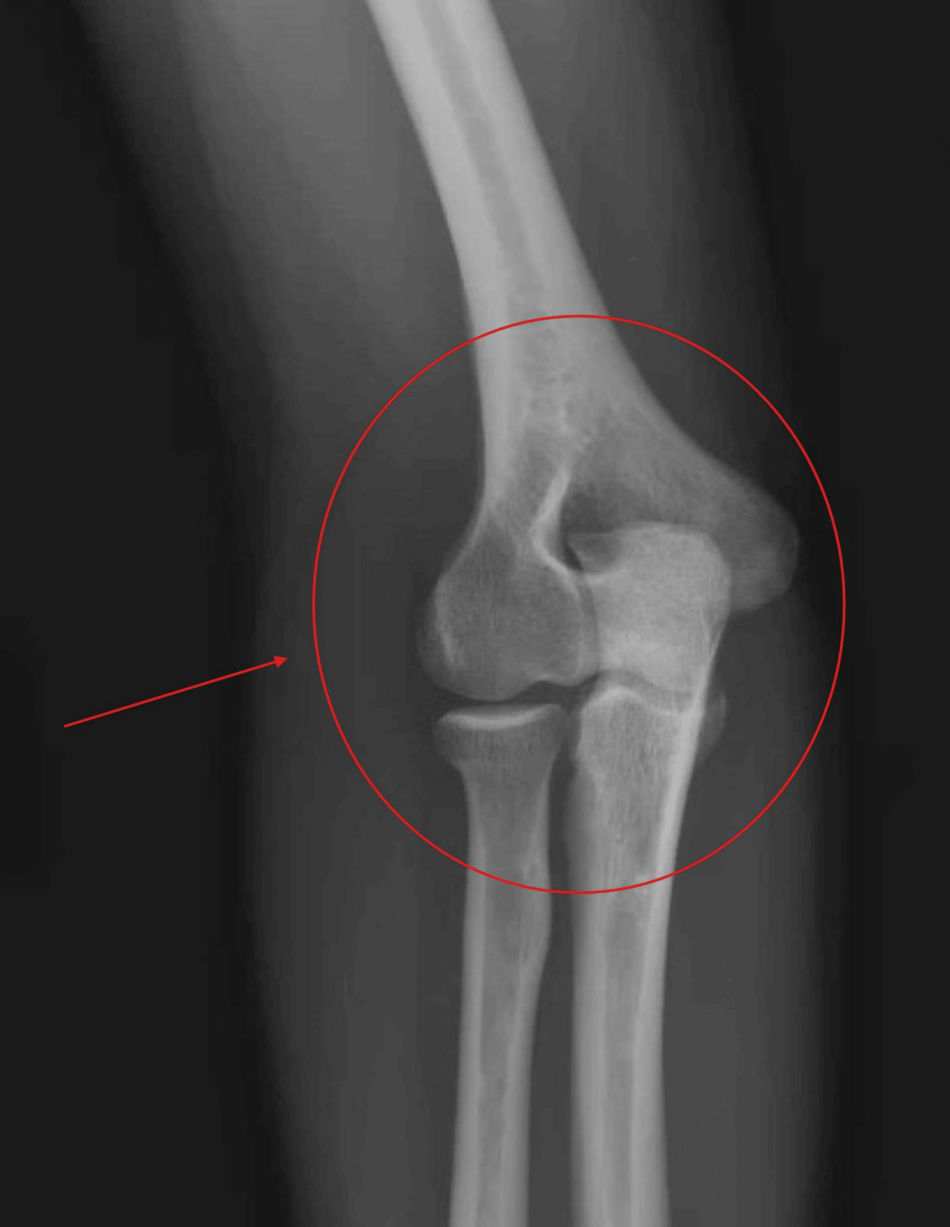
Anteroposterior radiograph of the right elbow showing normal osseous alignment The highlighted section demonstrates normal osseous alignment of the bone.

Plain radiographs of the right elbow (Figures 1, 2) demonstrated normal osseous alignment without degenerative or traumatic abnormality.

**Figure 3:**
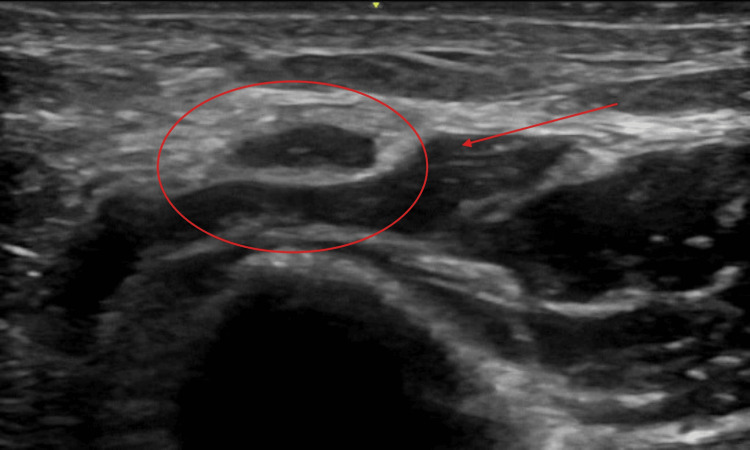
Sonographic evidence of ulnar nerve compression at the cubital tunnel Ultrasound imaging demonstrates focal enlargement of the ulnar nerve within the cubital tunnel, with increased cross-sectional area compared to proximal segments. The nerve appears hypoechoic with loss of the normal fascicular pattern, consistent with compressive neuropathy. Surrounding soft tissue structures contribute to the visible narrowing of the tunnel. Dynamic assessment with elbow flexion further demonstrates nerve subluxation and accentuation of compression, supporting a position-dependent etiology.

High-resolution ultrasound of the cubital tunnel demonstrated focal tenderness and dynamic impingement of the ulnar nerve at the medial epicondyle, suggestive of mechanical irritation during elbow movement. This is depicted in Figure 3.

Ultrasound is increasingly recognized as a useful adjunct in diagnosing focal nerve entrapment, particularly in cases with normal electrodiagnostic studies [5]. NCS of both upper extremities were normal, including median and ulnar motor and sensory studies. F-wave latencies were within normal limits. Needle EMG of sampled muscles showed no evidence of denervation or chronic reinnervation changes. Overall, there was no electrodiagnostic evidence of ulnar neuropathy or cervical radiculopathy. These findings are presented in Tables 1, 2, 3.

**Table 1:** Motor NCS APB: abductor pollicis brevis; ADM: abductor digiti minimi; B. elbow: below elbow; A. elbow: above elbow; NCS: nerve conduction study

Nerve/site	Muscle	Latency (ms)	Amplitude (mV)	Amp (%)	Duration (ms)	Distance (cm)	Lat diff (ms)	Velocity (m/s)	Temp (°F)
L median-APB									
Wrist	APB	2.94	9.2	100	5.8	6.5	-	-	96.7
Elbow	APB	6.79	8.9	96.8	5.7	23.5	3.85	61	-
R median-APB									
Wrist	APB	3.81	6.8	100	5.9	6.5	-	-	96.4
Elbow	APB	7.92	6.4	94.2	6	22.5	4.1	55	-
L ulnar-ADM									
Wrist	ADM	2.67	12.6	100	7.7	6.5	-	-	-
B. elbow	ADM	5.77	12.6	100.3	7.6	18.5	3.1	60	-
A. elbow	ADM	7.31	12.5	99.5	7.5	10.5	1.54	68	-
R ulnar-ADM									
Wrist	ADM	2.42	9.3	100	6.9	6.5	-	-	-
B. elbow	ADM	5.65	9	96.8	7	20.5	3.23	63	-
A. elbow	ADM	7.23	8.5	91.6	7.1	11	1.58	69	-
L median-ulnar lumbrical-interossei comparison									
Median wrist	Lumb II	3.29	2.1	100	6.7	10	-	-	-
Ulnar wrist	Interossei	2.98	4.6	222.5	5.4	10	-	-	-
Median-ulnar difference	-	-	-	-	-	-	0.31	-	-
R median-ulnar lumbrical-interossei comparison									
Median wrist	Lumb II	3.54	1.4	100	6.8	10	-	-	-
Ulnar wrist	Interossei	3.54	5	367.7	5.2	10	-	-	-
Median-ulnar difference	-	-	-	-	-	-	0	-	-

**Table 2:** Sensory NCS NCS: nerve conduction study

Nerve/study	Recording site	Onset latency (ms)	Peak latency (ms)	Negative peak amplitude (µV)	Segment	Distance (cm)	Peak diff (ms)	Velocity (m/s)
L median-digit II (antidromic)								
Wrist	Dig II	1.9	2.75	59.5	Wrist-dig II	13	-	69
R median-digit II (antidromic)								
Wrist	Dig II	2.15	3.08	60.6	Wrist-dig II	13	-	61
L ulnar-digit V (antidromic)								
Wrist	Dig V	1.88	2.56	54.7	Wrist-dig V	11	-	59
R Ulnar-digit V (antidromic)								
Wrist	Dig V	1.94	2.94	37.3	Wrist-dig V	11	-	57
L radial-anatomical snuff box (forearm)								
Forearm	Wrist	1.23	2.15	38.5	Forearm-wrist	10	-	81
R radial-anatomical snuff box (forearm)								
Forearm	Wrist	1.31	2.17	33.6	Forearm-wrist	10	-	76
L median-ulnar transcarpal comparison								
Median palm	Wrist	1.5	1.96	68.5	Median palm-wrist	8	-	53
Ulnar palm	Wrist	1.4	1.92	15.9	Ulnar palm-wrist	8	-	57
Median-ulnar difference	-	-	-	-	-	-	0.04	-
R median-ulnar transcarpal comparison								
Median palm	Wrist	1.46	1.94	42.7	Median palm-wrist	8	-	55
Ulnar palm	Wrist	1.44	1.92	13.5	Ulnar palm-wrist	8	-	56
Median-ulnar difference	-	-	-	-	-	-	0.02	-
L medial antebrachial cutaneous (forearm)								
Elbow	Forearm	2.15	2.63	7.6	Elbow-forearm	14	-	65

**Table 3:** F wave latencies APB: abductor pollicis brevis; ADM: abductor digiti minimi

Nerve	F lat (ms)	Normal (ms)
L median-APB	25.3	<31
R median-APB	27.7	<31
L ulnar-ADM	25.4	<32
R ulnar-ADM	26.7	<32

Although EMG/NCS are considered standard for diagnosing neuropathies such as cubital tunnel syndrome, their sensitivity decreases in cases of mild, intermittent, or dynamic compression [6]. Despite normal electrodiagnostic studies, the patient was diagnosed with right cubital tunnel syndrome, supported by both clinical findings and ultrasound, and post-surgical neuropathic irritation following thoracic outlet decompression.

**Figure 4:**
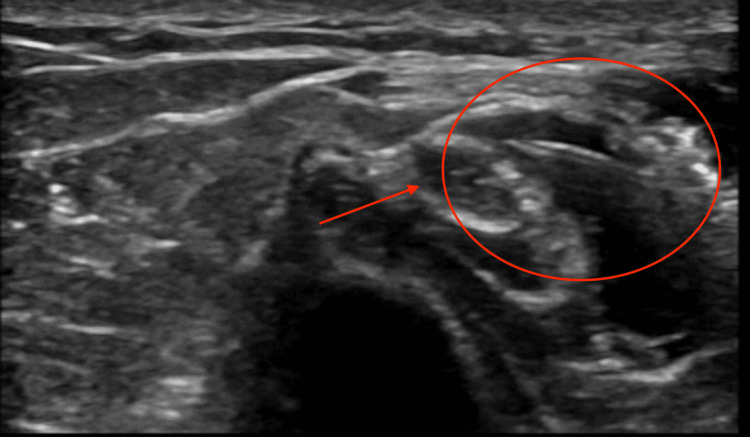
Ultrasound-guided hydrodissection of the ulnar nerve at the cubital tunnel Ultrasound imaging depicts the injection of dextrose and dexamethasone with subsequent hydrodissection of the ulnar nerve away from surrounding tissues.

Conservative management involved the use of gabapentin and naproxen, modified activity with avoidance of prolonged elbow flexion, night splinting for cubital tunnel protection, and nerve gliding exercises incorporated into physical therapy. The patient underwent an ultrasound-guided ulnar nerve hydrodissection in the cubital tunnel. The injectate included dextrose solution, lidocaine, and corticosteroid in the form of dexamethasone. The ulnar nerve was visualized under ultrasound, and hydrodissection was performed at the proximal, focal impingement, and distal segments of the cubital tunnel. This is depicted in Figure 4.

Hydrodissection is proposed to mechanically separate the nerve from surrounding fascial constraints and improve nerve gliding, with emerging evidence supporting symptomatic improvement in entrapment neuropathies [7]. The procedure was well tolerated without complications.

The immediate post-procedural response included local anesthesia effect with reduced tenderness. The patient was scheduled for follow-up at six weeks to assess symptom progression and consider repeat hydrodissection or surgical decompression if symptoms persisted.

## Discussion

This case highlights several important clinical and diagnostic considerations in upper extremity neuropathic syndromes. Although EMG/NCS remain gold standard tools for diagnosing cubital tunnel syndrome, their sensitivity is imperfect. Early or mild compression may not demonstrate conduction slowing or denervation changes [6,8]. Dynamic compression occurring only during elbow flexion may also evade detection in standard resting studies. The patient had clear clinical symptoms and ultrasound evidence of nerve impingement despite normal electrodiagnostics, consistent with prior literature showing false-negative rates in early ulnar neuropathy [3].

High-resolution ultrasound allows real-time visualization of nerve morphology, mobility, and dynamic compression. It has been shown to detect focal enlargement or impingement even when EMG is normal [5,9]. In cubital tunnel syndrome, ultrasound may demonstrate nerve flattening, hypoechogenicity, or subluxation. In this case, ultrasound confirmed mechanical impingement at the cubital tunnel, supporting the clinical diagnosis. Additionally, ultrasound offers the advantage of bedside dynamic assessment, allowing evaluation of nerve behavior during elbow flexion and extension, which is particularly relevant in patients with intermittent or activity-dependent symptoms that are not reproducible in static electrodiagnostic testing.

Thoracic outlet decompression alters brachial plexus tension and upper limb biomechanics. While often beneficial, it may unmask distal compressive neuropathies or contribute to altered nerve glide patterns [4]. Neural sensitization following chronic compression may also persist despite surgical decompression. Furthermore, postoperative changes in posture, muscular balance, and scapulothoracic mechanics can shift load distribution along the entire neural axis, potentially increasing susceptibility to distal entrapment sites such as the cubital tunnel.

The patient’s symptom onset immediately postoperatively suggests either unmasking of pre-existing subclinical cubital tunnel compression, iatrogenic alteration in nerve tension dynamics, or post-surgical inflammatory neuropathy. Another consideration is central and peripheral sensitization following prolonged preoperative neural compromise, which may lower the threshold for symptom perception even in the absence of significant structural worsening. The coexistence of improved proximal symptoms with the emergence of distal neuropathic pain supports the concept of “double-crush” physiology, in which proximal decompression may reveal latent distal entrapment.

Perineural hydrodissection is an emerging technique aimed at separating nerves from surrounding adhesions through fluid injection. Proposed mechanisms include mechanical decompression, improved vascularity, and reduction of perineural inflammation [7,10]. It may also restore nerve excursion within the fascial planes, which is increasingly recognized as a key component of normal neural biomechanics. Small clinical studies suggest benefit in entrapment neuropathies, though high-quality randomized trials remain limited [11]. The addition of corticosteroid and dextrose may provide combined anti-inflammatory and regenerative effects, although the relative contribution of each component remains unclear.

Importantly, this case underscores the need for a multimodal diagnostic approach when clinical suspicion remains high despite negative electrodiagnostic testing. Integrating clinical examination, provocative testing, and high-resolution ultrasound may improve diagnostic yield and better guide management decisions.

This case emphasizes that normal EMG does not exclude clinically significant cubital tunnel syndrome; ultrasound is a valuable complementary diagnostic tool; post-surgical neuropathic symptoms require a broad differential; and both conservative and interventional therapies may be appropriate even without electrodiagnostic confirmation. Surgical decompression remains a consideration for refractory cases, though careful correlation with imaging and clinical findings is essential [12].

## Conclusions

Cubital tunnel syndrome can present with significant clinical symptoms despite normal electrodiagnostic testing, particularly in post-surgical or early-stage cases where compression is dynamic or mild. This highlights the limitations of relying solely on EMG and NCSs and reinforces the importance of a thorough clinical examination. High-resolution ultrasound serves as a valuable adjunct by enabling real-time, dynamic assessment of the ulnar nerve, identifying structural abnormalities or focal compression not detected on electrodiagnostics. In this context, ultrasound-guided hydrodissection may represent a minimally invasive therapeutic option that has been reported to help alleviate symptoms by reducing perineural adhesions and improving nerve mobility, particularly in patients with postoperative scarring. Overall, a multimodal approach integrating clinical evaluation, imaging, and targeted interventions is essential for accurate diagnosis and effective management of complex neuropathic conditions.

## References

[1] Caliandro P, La Torre G, Padua R, Giannini F, Reale G, Padua L (2025). Treatment for ulnar neuropathy at the elbow. Cochrane Database Syst Rev.

[2] Bozentka DJ (1998). Cubital tunnel syndrome pathophysiology. Clin Orthop Relat Res.

[3] Campbell WW (2008). Evaluation and management of peripheral nerve injury. Clin Neurophysiol.

[4] Illig KA, Donahue D, Duncan A (2016). Reporting standards of the Society for Vascular Surgery for thoracic outlet syndrome. J Vasc Surg.

[5] Thain LM, Downey DB (2002). Sonography of peripheral nerves: technique, anatomy, and pathology. Ultrasound Q.

[6] Beekman R, Visser LH (2004). High-resolution sonography of the peripheral nervous system-a review of the literature. Eur J Neurol.

[7] Lam KH, Yoon Y, Su DC, Suryadi T, Suhaimi A, Wu YT, Tsan Y (2026). Ultrasound-guided hydrodissection for peripheral neuropathy: an evidence-based intervention whose time has finally come. Cureus.

[8] Campbell WW, Carroll C, Landau ME (2015). Ulnar neuropathy at the elbow: five new things. Neurol Clin Pract.

[9] Martinoli C, Bianchi S, Gandolfo N, Valle M, Simonetti S, Derchi LE (2000). US of nerve entrapments in osteofibrous tunnels of the upper and lower limbs. Radiographics.

[10] Lleva JMC, Munakomi S, Sun CE, Chang KV (2026). Ulnar Neuropathy. https://www.ncbi.nlm.nih.gov/books/NBK534226/.

[11] Dellon AL (1989). Review of treatment results for ulnar nerve entrapment at the elbow. J Hand Surg Am.

[12] Nakashian MN, Ireland D, Kane PM (2020). Cubital tunnel syndrome: current concepts. Curr Rev Musculoskelet Med.

